# Early adiposity rebound: predictors and outcomes

**DOI:** 10.1186/s13052-024-01671-4

**Published:** 2024-05-15

**Authors:** Alessandra Li Pomi, Giorgia Pepe, Tommaso Aversa, Domenico Corica, Mariella Valenzise, Maria Francesca Messina, Letteria Anna Morabito, Stefano Stagi, Malgorzata Wasniewska

**Affiliations:** 1 Department of Human Pathology of Adulthood and Childhood, University of Messina, Via Consolare Valeria, 98124 Messina, Italy; 2 Pediatric Unit “G. Martino University Hospital, Messina, Italy; 3 Department of Health Sciences, University of Florence, Florence, Italy; 4 Meyer Children’s Hospital IRCCS, Florence, Italy

**Keywords:** Adiposity rebound, Early adiposity rebound, Obesity, Obesity comorbidities, Sexual dimorfism

## Abstract

Adiposity rebound (AR) refers to the second rise of the body mass index (BMI) curve that usually occurs between six and eight years of age. AR timing has a significant impact on patients’ health: early AR (EAR), usually before the age of five, is considered to be the earliest indicator of obesity and its related health conditions later in life. Many studies have evaluated factors that can be predictors of EAR, and identified low birth weight and gestational weight gain as novel predictors of EAR, highlighting the role of the intrauterine environment in the kinetics of adiposity. Furthermore, children with breastfeeding longer than 4 months have been found to be less likely to have an EAR, whereas children born to advanced-age mothers, high maternal BMI had a higher risk of having an EAR. Some differences were found in the timing of AR in boys and girls, with girls being more likely to have EAR. The aim of this review is to answer the following three questions: 1) Which are the prenatal and perinatal factors associated with increased risk of EAR? Is gender one of these? 2) Which are the outcomes of EAR in childhood and in adulthood? 3) Which measures can be taken in order to prevent premature AR?

## Introduction

The term “adiposity rebound” (AR) refers to the second rise of body mass index (BMI) which occurs between the age of 6 and 8 years. BMI increases during the first year of life and then decreases reaching a nadir around 6 years of age. The point of minimal BMI value (the nadir of the BMI curve) is the beginning of the AR. Thereafter, it increases again gradually during childhood, adolescence and most of adulthood [1, 2]. The highest point of the BMI trajectory in infancy is called “adiposity peak” (AP) [3]. Whereas, early adiposity rebound (EAR) can be diagnosed if the BMI curve reaches its lowest point before five years of age [4].

BMI is the best tool for assessing nutritional status, but there are also other measurement possibilities such as skin folds and body composition in terms of fat mass (FM) to assess body fat, fat-free mass (FFT) and body lean mass [2, 5]. Body lean mass includes the weight of the internal organs, bones, muscles, water ligaments, tendons, central nervous system and bone marrow, which contain a small percentage of essential fat. On the other hand, FFT does not include any fat and is calculated by subtracting body fat weight from total body weight. Nevertheless, BMI remains the most practical parameter in daily activity for the evaluation of nutritional status and its changes.

The role of fat and lean mass in AR has been re-evaluated. Initially, the onset of AR was attributed to an increase in fat mass, but more recent research has suggested coincidence of AR with an interruption of fat mass decline and an increase in lean mass. In this context, an increase in fat mass might actually lag 2–3 years behind the increase in lean mass, particularly for boys. Therefore, a more accurate term for AR could be “BMI rebound” [6]. It is also true that a higher BMI could reflect not only increased weight, but also relatively low height. However, in this context, early rebound reflects greater weight gain rather than slower height acquisition, with the weight gain being predominantly due to a rapid elevation in the deposition of body fat [7].

EAR is considered the earliest indicator for obesity and its related health conditions later in life.

A relatively recent American study has shown that each year decrease in age at AR was associated with a 2.5kg/m2 increase in the predicted BMI level at around 20 years of age [8].

Moreover, EAR was associated with advancement of skeletal maturity [9].

**Fig. 1:**
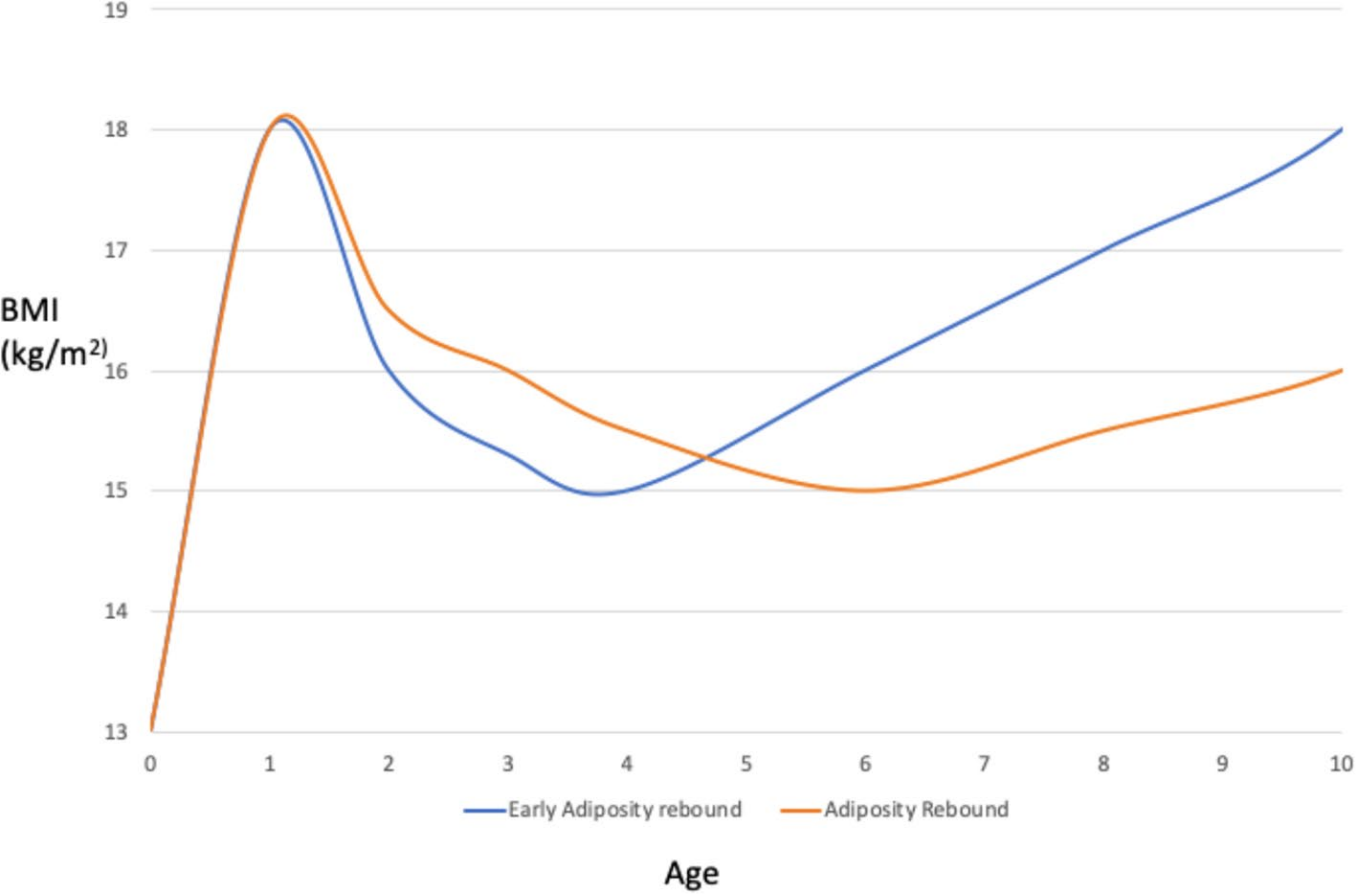
Early adiposity rebound and normal adiposity rebound. The figure shows the different growth patterns in EAR and in physiological AR. Compared to normal AR, EAR is associated with low fatness before the rebound and high fatness from the time of AR, with a high BMI at adult age but normal or even low BMI level before the rebound

The importance of assessing the age at AR is to study the growth pattern, more than the absolute fatness level (Fig. 1). In most obese patients EAR is recorded, suggesting that determinants of obesity start to play a role in youth. In particular, EAR is associated with low fatness before the rebound and high fatness from the time of AR, with a high BMI at adult age but normal or even low BMI level before the rebound [2] (Fig. 1). This condition is associated with insulin resistance and coronary heart diseases later in life [2]. Similarly, late AP influences the growth pattern and represents an indicator for future obesity-related consequences [3].

The early recognition of auxological parameters that can predict the future risk of obesity is mandatory in order to avoid the development of obesity later in life. The most effective strategy for this disease is prevention rather than treatment.

## Materials and methods

All Authors of this narrative review are part of the scientific group that deals with pediatric endocrinology of the Paediatric Clinic of the University of Messina and University of Florence.

They focused their search on the predictors and outcomes on EAR in childhood in the context of existing literature data. They were aimed at finding the answers to three questions: 1) Which are the prenatal and perinatal factors associated with an increased risk of EAR? Is gender one of these? 2) Which are the outcomes of this early metabolic alteration in childhood and in adulthood? 3) Which measures can be taken in order to prevent premature AR?

The authors performed a literature search in PubMed, using selected key words (‘Adiposity rebound OR “early adiposity rebound’) AND (‘children OR childhood’) AND (‘predictors OR outcome’) AND (‘obesity OR overweight’). In addition to the automated search, a manual search for additional relevant publications within the bibliographies of the papers previously automatically identified was made.

All authors independently identified the most relevant papers published in English in the past 15 years (arbitrarily established by them), including original papers, metanalyses, clinical trials, and reviews. Case reports, series and letters were excluded.

The contributions were critically reviewed and collected by all the authors, and each approved the final version.

## Results and discussion

### Which are the prenatal and perinatal factors associated with an increased risk ok EAR? Is gender one of these?

Nowadays AR occurs earlier compared to several decades ago [10]. This could be explained by the current obesogenic environment characterized by reduction in physical activity and increase in energy intakes since the very early years of life [11]. In this context, the increasingly precocious exposure to technological devices certainly plays a role [12, 13].

**Fig. 2:**
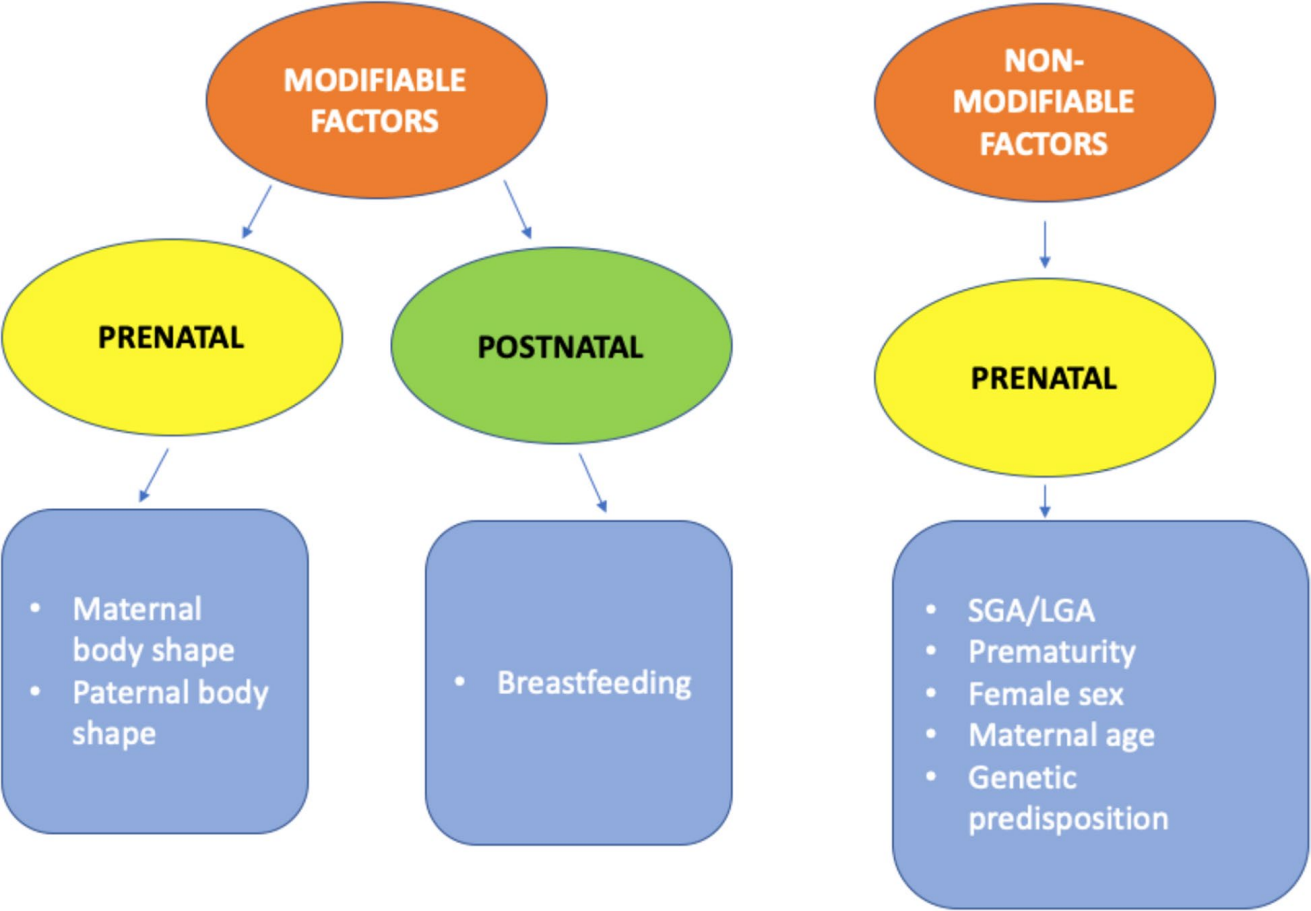
Factors influencing the timing of Adiposity Rebound (AR). On the left, the figure shows modifiable factors influencing the time of AR, that can be divided into prenatal, which is parents body shape, and postnatal, where breastfeeding is the only factors being associated with a reduced risk of EAR. On the right, the figure shows non modifiable factors influencing the time of AR, which are related to the pregnant woman and gestation

On the other hand, several studies have focused their research on a set of early prenatal and postnatal determinants of age at AR (Fig. 2). First of all, birth weight is one of the most studied factor that influence future metabolic state. It is known that not only a low birth weight typical of small for gestational age (SGA) newborns but also a large for gestational age (LGA) birth weight is associated with adverse health outcomes during adulthood [12]. In particular, the amount of FM and FFM at the age of 5-7 years in children who were born SGA or LGA was investigated in the German study conducted by Gatiens et al. [14]. They showed that in LGA children, a compensatory catch-down postnatal growth might be a risk factor for the development of disproportionate gain in fat over lean mass in terms of fatty mass index (FMI), whereas in children born SGA, a catch-down postnatal growth seems to favor the subsequent accretion of lean over fat mass. Nevertheless, catch-down postnatal growth led to a lower BMI at the age of 5–7 years compared to rapid postnatal growth [14]. On the other hand, they found in boys a higher propensity of lean mass accretion during postnatal catch-up growth compared to girls [14]. Finally, Cissé et al. also identified birth weight for gestational age (GA) as a predictor of age at AR. In this context, they found that SGA children had an increased risk of EAR, whereas children born LGA did not [1]. This data is confirmed by the study conducted by Lin et al., which is discussed below [3]. In contrast, the Italian study conducted by Baldassare et al. on preterm infants found a correlation between EAR and being LGA [4]. So, the influence of birth weight correlated to GA on the timing of AR is still controversial.

On the other hand, GA alone is an independent risk factor that could influence the growth pattern. Specifically, it is known that children born preterm usually present a catch-up postnatal growth, regardless of birth weight.

In the Chinese study conducted by Lin et al., children born preterm were found to have higher risk of late AP, after 12 months of age. They hypothesized that this longer time left for BMI increase might contribute to a larger BMI value later in life [3].

Returning to the main theme of this review, Baldassarre et al. from Italy have studied the prevalence of EAR in preterm infants, assessing it at 54%. Furthermore, they found that ex-preterm infants with EAR had a significantly higher BMI at the age of 7 years, compared to those with regular AR. Overall, the prevalence of obesity in subjects with EAR was higher, but the difference was not statistically significant [4].

Moreover, in Japan Maeyama et al. simultaneously studied the potential GA dependency in SGA children on BMI trajectory during the first 3 years of life and whether SGA children were at risk for AR depending on GA, finding that 7.1% of SGA children had EAR. They also found that the mean BMI SDSs for SGA children <36 weeks of GA increased rapidly in the first 4 months of life of around 1.4 SDS, then stayed stable [15].

Other modifiable pre-birth or early-life factors associated with the timing of AP or AR were evaluated by the Chinese study conducted by Lin et al., studying a population of children born after 2010 and who grew up in a metropolis of China. Using univariate regression analysis, they found that sex and gestational age were factors associated with timing of AP. Specifically, females were more likely to have late AP than males, and preterm infants were at higher risk of late AP [3].

Girls present worse outcomes also in term of EAR: in their longitudinal study, Aris et al. evaluated the kinetics of BMI curve, and found that girls had earlier adiposity rebound compared to boys [16].

This data has been confirmed by the recent systematic review by Zhou et. al, which included 28 studies up to August 2021, showing an overall prevalence of EAR of 40%, 5% higher in girls than in boys, with the former achieving AR on average 3.38 months before the latter [17].

To summarize, literature data seems to give a worse outcome in terms of adiposity in females than in males.

Others have focused their interest on maternal factors as predictors of EAR: in the long-term retrospective study, Péneau et al. reported that lower age at AR was associated with larger maternal body shape in both sexes and with paternal body shape in girls only [18]. Dorosty et al, meanwhile, found that maternal BMI had an impact on AR in men and paternal BMI in women [10].

Similarly, Ip et al. in their study found that mothers of early rebounders were 3 units of BMI heavier. Moreover, in multivariate analysis, also poor maternal education was a predictor of EAR [6].

For Cissè et al., the association between parental BMI and age at AR does not depend on risk-allele score for obesity. Indeed, parental BMI reflects the familial environment, which could be obesogenic [1]. To conclude, parents’ metabolic state undoubtedly influences their children, as demonstrated by the literature.

Another factor that can be attributed to the mother is breastfeeding. Lin et al. showed that children with breastfeeding longer than 4 months were less likely to have an early AR compared to the others. Moreover, children who were born to advanced-age mothers (>35 years) had higher risk of EAR [3].

Few studies have evaluated if physical activity and dietary intake may influence the age of AR, studying children at the age at which adiposity rebound typically occurs. However, no studies appear to have directly assessed whether these environmental factors have any impact on the timing of adiposity rebound [11].

The cited study conducted by Ip et al. showed that increased caloric intake is a significant predictor of EAR, so limiting excess calories could delay premature AR and lower the risk of future obesity [6].

Similarly, few studies have evaluated the possible genetic role in the timing of AR. It is known that human growth is strongly influenced by genetics and children with early adiposity rebound often have obese parents. Nevertheless, the role of heritability has not yet been accurately defined.

The cited study conducted by Cissè et al. has found that the genetic susceptibility to obesity was associated with an increased probability of EAR, which is the result of a cumulative effect of individual different SNPs [1].

Moreover, genetic differences might influence sex differences in physical development during infancy. As Lin et al. found, females are at higher risk of being overweight or obese at any stage of life [3].

Therefore, congenital hypothyroidism (CHT) and congenital adrenal hyperplasia (CAH) are the most studied paediatric diseases associated with EAR. It is well known that thyroid hormones play a fundamental role in physiological growth and that the condition of CHT can impact on normal height and weight development. Several studies have evaluated the association of CHT with the rate of obesity and the timing of AR. In Taiwan, Chan et al. have found that children with CHT might have an higher risk of obesity due to AR occurring at an earlier age than the general population, with lower T4 levels after treatment compared to non-obese population. Specifically, a total prevalence (boys and girls) of obesity of 32.2% was reported in children with CHT, and an earlier mean age at AR in girls with CHT compared to non-obese female population. Moreover, the mean age of AR in obese/overweight girls with CHT was also lower than in girl of the general population with a BMI in the 97^th^ percentile (3.17 +/- 1.38 vs. 3.92 years old) [19].

Similarly, the English study by Wong et al. showed that EAR (before the age of 49 months) was more frequent in children with CHT compared to healthy controls (54.7% vs 21.4%), with no significant relationships between timing of AR and initial thyroid function or age at normalization of TSH [20].

On the other hand, focusing on the long-term prognosis of CHT, Livadas et al. found that the elevated BMI values characterizing early childhood returned to normal ranges in adolescence and the BMI decrease to the nadir is less pronounced [21].

Another paediatric disease found to have a relationship with EAR, with risk of future obesity, is CAH due to 21-hydroxylase deficiency (21-OHD) [22]. Takishima et al., in their 21-OHD population, found that the risk of EAR could not be reduced by adjusting the glucocorticoid therapy and that the metabolic status in these patients might be affected by some fetal factors, such as birth body size (lower body weight and lower BMI) [22].

The same disease was studied by Bhullar et al., finding EAR to predict future cardiometabolic disease in obese patients [23].

### Which are the outcomes of EAR in childhood and in adulthood?

The timing of AR is an independent predictor of obesity in later childhood and adulthood. In detail, an EAR increases the risk of being overweight/obese and of being affected by other obesity-related comorbidities, such as impaired glucose tolerance, type 2 diabetes and cardiovascular disorders in childhood, adolescence and adulthood [1, 3, 11].

Also AP has predictive significance for these adverse health outcomes [3].

Certainly, this parameter is secondary for importance compared to the timing of AR. In fact, few studies evaluated the association between the timing of AP and risk of being overweight or obese. With the same population data by which were evaluated factors influencing the development of EAR, Lin et al. aimed to identify the association between the timing of AP and AR and the risk of developing overweight or obesity in first‐grade school children, finding that children with late AP or EAR were at risk of overweight but not obesity. Nevertheless, there are prevention strategies that can reduce the risk of these conditions, such as physical activity in children with a late AP and limitation of screen time in those with EAR [24].

Returning to EAR, several studies have investigated the relationship between the timing of AR and future occurrence of metabolic consequences. First of all, Koyama et al. in Japan studied the presence of metabolic syndrome at the age of 12 years old through a longitudinal population-based study, finding that children with EAR were predisposed to future development of metabolic syndrome [25]. Specifically, they found that an AR before 4 years of age was associated with alterations of the lipid profile: elevated triglycerides, apolipoprotein B and atherogenic index. Moreover, some sex differences have emerged from the study: at 12 years of age, boys had low high-density lipoprotein cholesterol and elevated blood pressure, whereas girls had elevated apolipoprotein B.

On the other hand, the long-term retrospective study carried out by Peneau et al. has found an higher body mass index and waist circumference at adulthood in men and women with an earlier AR [18]. Moreover, women with EAR had significantly higher triglyceride, low-density lipoprotein-cholesterol, systolic and diastolic blood pressure at adulthood. In men, timing of AR was not significantly associated with any of these cardiometabolic risk factors [18].

Various historical studies by Rolland-Cachera have studied adiposity rebound, finding for example that EAR was associated with higher BMI or subscapular skin fold at age 21 years [26] and advanced skeletal maturity [27].

Similarly, Taylor et al. have studied children between 5 and 9 years, finding a causal relationship between BMI in AR period and alteration in body fat assessed by dual X-ray absorptiometry (DXA) rather than by alteration in lean mass. Specifically, children undergoing EAR gained fat at a faster rate than children who rebounded at a later age, justifying the use of the term ‘AR’ rather than ‘BMI rebound’ [7].

Going back to the already discussed conditions associated with EAR, such as prematurity and being SGA, we need to underline that they can influence the nutritional status also during childhood and adulthood.

Firstly, in preterm babies, the risk for future obesity and obesity-related complication seems to be linked to the cited “catch-up growth” that occurs in the early ages of life, causing rapid weight gain and early increase in fat mass and BMI [4].

This is due to the energy restriction in utero, favouring the development of important organs, such as brain and heart, at the cost of other organs such as muscular tissue and endocrine pancreas. Moreover, they develop an early accretion of FM over FFM, contributing to inflammation and frailty in the future [12].

In these terms, prematurity is and independent risk factor for developing cardiovascular disease in adolescence and type 2 diabetes in adulthood [3].

Secondly, in children born SGA or simply with low birth weight, an increased incidence of obesity and future coronary heart disease and type 2 diabetes was found, suggesting that these conditions reflect a lifelong health disadvantage [13].

On the other hand, it is known that the metabolic status is strictly related to the pubertal development. In this context, Marakaki et al. have evaluated the relationship between the timing of AR and the presence of premature adrenarche (PA), which means a pubarche that occurs before the age of 8 in girls and of 9 in boys. Studying the growth pattern and the role of adiposity in PA, they found that AR occurred earlier in both boys and girls with PA compared to controls, with a difference of 1.65 and 1.2 years respectively. Moreover, children with PA were taller and heavier than controls since early life. Finally, obese girls with PA had greater levels of Dehydroepiandrosterone-sulfate (DHEAS) and earlier age at AR than in non-obese girls with PA [28]. The exact underlying pathogenetic mechanism is not fully known yet. Probably, a mild hyperactivity of the hypothalamic-pituitary-adrenal (HPA) gland causes an excessive secretion of adrenocorticotropic hormone which stimulates the adrenal gland to produce androgens, such as DHEAS [28].

In conclusion, early alterations in the metabolic state, such as EAR, have long-term consequences demonstrated in literature and previously explained. However, not less important is the social impact that a condition of early obesity and its related diseases can have for the whole life of these children. Being overweight or obese often has a negative connotation not only for the resulting health problems, but there may be a certain discrimination against obese subjects as they are not considered aesthetically pleasing, causing weight stigma.

### Which measures can be taken in order to prevent premature AR?

From analysing the literature, we can distinguish modifiable and non-modifiable prenatal and perinatal factors for EAR. Birth weight, gestational age, genetic susceptibility and sex belong to the group of non-modifiable factors. For this reason, the only future perspective could be the introduction of early measures to ensure an adequate caloric intake for the child since birth, paying more attention especially in the period which precedes the AR. In this regard, early identification of subjects at risk of EAR is fundamental, in order to deal with the adaptive metabolism that occurs after a period of energy deficit in early stages of life.

A particular attention should be paid to SGA population, in order to avoid overnutrition and excessive protein intake that can potentiate or accelerate catch-up growth [29].

Regarding modifiable factors, the mother’s role is undoubtedly crucial. Eradication of risk factors, such as high maternal BMI before pregnancy, is part of the project focused on the importance of nutrition in the first 1000 days. Furthermore, ensuring adequate counselling of breastfeeding could favour the continuation of the latter for longer time. Breastfeeding is a natural and effective strategy to guarantee adequate nutrition to the infant, which combined with healthy oral feeding represents the best strategy in the first year of life. Thereafter, the family, together with the family paediatrician, have to devote time to the establishment of good eating and living habits. Together with longer breastfeeding, later main meal food introduction and use of home-made foods is inversely associated with the risk of faster weight gain and EAR [30].

Moreover, social determinants of health play a role in preventing the development of metabolic disorders. In these terms, it is important to promote the practice of unplanned physical activity, for example encouraging to go to school by foot and to encourage movement even at home could be the first strategy to lay the foundations for future metabolic health. Children should learn from family and from school which are the examples to follow in terms of body weight, in order to have a right control over their own. Despite every country has different levels of governmental and societal support, a more systematic organization in order to give a continuous help to individual choices and challenges would be necessary.

## Conclusions

Our research on literature data confirms that the modality of AR is related to future health conditions. EAR is a useful marker to predict future obesity and its correlated comorbidities in paediatric population. In terms of AR, a certain degree of sexual dimorphism has been found, being female sex more likely to develop precocious AR. Modifiable environmental risk factors for EAR should be soon recognized in order to suggest new approaches to improve prevention strategies starting from early life. Therefore, monitoring of the timing of AR may be an effective method for early identification of children at risk for metabolic alterations. For this reason, family paediatricians should perform a proper follow-up on height-weight growth curves of their patients in order to deploy prevention strategy in case of an acceleration of growth rate. The auxologic evaluation should periodically include also the BMI curve for the debated concept of “BMI rebound”. The eventual presence of EAR should be considered as a predictor of future obesity and its comorbidities.

## Data Availability

All studies and data analysed during this study are included in this article. Further enquiries can be directed to the corresponding author.

## Abbreviations

AR: Adiposity rebound
BMI: Body mass index
EAR: Early adiposity rebound
AP: Adiposity peak
FM: Fatty mass
FFM: Fat-free mass
SGA: Small for gestational age
LGA: Large for gestational age
GA: Gestational age
CHT: Congenital hypothyroidism
21-OHD: 21-hydroxylase deficiency
DXA: Dual X-ray absorptiometry
PA: Premature adrenarche
DHEAS: Dehydroepiandrosterone-sulfate
HPA: Hypothalamic-pituitary-adrenal
